# Development of robust isothermal RNA amplification assay for lab-free testing of RNA viruses

**DOI:** 10.1038/s41598-021-95411-x

**Published:** 2021-08-06

**Authors:** Radhika Biyani, Kirti Sharma, Kenji Kojima, Madhu Biyani, Vishnu Sharma, Tarun Kumawat, Kevin Maafu Juma, Itaru Yanagihara, Shinsuke Fujiwara, Eiichi Kodama, Yuzuru Takamura, Masahiro Takagi, Kiyoshi Yasukawa, Manish Biyani

**Affiliations:** 1 Department of Bioscience and Biotechnology, Japan Advanced Institute of Science and Technology, 1-1 Asahidai, Nomi City, Ishikawa 923-1292 Japan; 2 BioSeeds Corporation, JAIST Venture Business Laboratory, Ishikawa Create Labo, Asahidai 2-13, Nomi City, Ishikawa 923-1211 Japan; 3 Division of Food Science and Biotechnology, Graduate School of Agriculture, Kyoto University, Sakyo-ku, Kyoto 606-8502 Japan; 4 Biyani BioSolutions Pvt. Ltd., Biyani Group of Colleges Venture Business Laboratory, R-4, Sector 3, Vidhyadhar Nagar, Jaipur, 302039 India; 5 Department of Developmental Medicine, Research Institute, Osaka Women’s and Children’s Hospital, 840 Murodocho, Izumi, Osaka 594-1101 Japan; 6 Department of Biosciences, School of Biological and Environmental Sciences, Kwansei-Gakuin University, 2-1 Gakuen, Sanda, Hyogo 669-1337 Japan; 7 Division of Infectious Diseases, International Research Institute of Disaster Science, Tohoku University, 2-1 Seiryocho Aoba-ku, Sendai, Miyagi 980-8575 Japan

**Keywords:** Biomedical engineering, Synthetic biology, Nucleic acids

## Abstract

Simple tests of infectiousness that return results in minutes and directly from samples even with low viral loads could be a potential game-changer in the fight against COVID-19. Here, we describe an improved isothermal nucleic acid amplification assay, termed the RICCA (RNA Isothermal Co-assisted and Coupled Amplification) reaction, that consists of a simple one-pot format of ‘sample-in and result-out’ with a primary focus on the detection of low copy numbers of RNA virus directly from saliva without the need for laboratory processing. We demonstrate our assay by detecting 16S rRNA directly from *E. coli* cells with a sensitivity as low as 8 CFU/μL and RNA fragments from a synthetic template of SARS-CoV-2 with a sensitivity as low as 1740 copies/μL. We further demonstrate the applicability of our assay for real-time testing at the point of care by designing a closed format for paper-based lateral flow assay and detecting heat-inactivated SARS-COV-2 virus in human saliva at concentrations ranging from 28,000 to 2.8 copies/μL with a total assay time of 15–30 min.

## Introduction

With the emergence of new infectious virus strains that result in outbreaks, the identification and isolation of infected suspects (symptomatic or asymptomatic) constitute the most effective method for preventing human-to-human disease transmission and pandemics^1,2^. In this stream, a simple yet reliable, rapid and field-deployable molecular diagnostic tool for the detection of nucleic acid sequences of infectious viruses is vital for the massive screening of the population and has been greatly needed worldwide during the current global wave of coronavirus disease 2019 (COVID-19). Hundreds of molecular tests have been rapidly introduced but still face an arduous journey to reach mass usage, due mainly to the shortcomings in the existing laboratory-based testing paradigm for RNA-based viral diagnostics^3^. First, nucleic acid isolation from clinical samples, which is routinely performed in a laboratory for PCR testing, a current gold-standard diagnostic test, constitutes a major bottleneck. This bottleneck becomes more challenging for RNA-based viruses because rapid RNA degradation contributes to poor clinical sensitivity^4^. Second, the requirements of bulky expensive instrumentation, such as thermal cyclers with fluorometry, hinder the application of current PCR testing in the field.

To obtain PCR-similar molecular testing outside a centralized laboratory, various isothermal (single-temperature) nucleic acid amplification methods have been devised and are in a continuous race to achieve a performance similar to that of PCR tests. The major candidates include recombinase polymerase amplification (RPA)^5–9^, nucleic acid sequence-based amplification (NASBA) or RNA-specific amplification^10–12^, and loop-mediated isothermal amplification (LAMP)^13^. The advantage of isothermal amplification methods over PCR is that they do not require bulky instrumentation, enable rapid (10–60 min) amplification of nucleic acids at a constant temperature (e.g., 37–65 °C) and therefore improve throughput in situations in which large numbers of clinical samples must be processed and facilitate point-of-care diagnosis. Recent advances in saliva-based isothermal amplification-based assays, such as RT-LAMP or RT-RPA, have encouraged the testing of SARS-CoV-2-containing clinical samples^14–17^. Although encouraging, isothermal amplification methods have also been shown to produce more false-positive (i.e., lower test specificity due to nonspecific amplification) results than PCR testing^18^. Specifically, false-positive signals in RPA, which are more likely to occur due to nontarget-triggered amplification reactions or primer-dependent artifacts, are often exacerbated at low reaction temperatures and are magnified with increases in the reaction time^19^. To circumvent this issue, the application of probe chemistry^20^, portable gel electrophoresis^21^, and CRISPR-based technology^22,23^ has been demonstrated to improve the performance of RPA. Noticeably, the problem of nonspecific amplification in RPA generally occurs when the viral loads in the tested samples are low^24^. Additionally, if the target is RNA, the amplification methods depend on the efficiency of the initial molecular step, reverse transcription, which can greatly affect post DNA-specific amplification efficiency at low concentrations of the RNA template^25^ and has also been reported to have an effect of apparent false-negative results (from 17% to as high as 48%) due to the role of the SARS-CoV-2 viral load dynamics^26^. Therefore, to maintain the integrity of RNA in the reaction sample, a method to support reverse transcription and effectively amplify a low viral RNA load is crucial.

RNA-specific amplification specifically amplifies a target RNA sequence at a temperature near 40 °C with reverse transcriptase (RT) and RNA polymerase^10–12^. In RNA-specific amplification, RT synthesizes promoter-bearing double-stranded (ds) DNA with the help of its RNase H activity. RNA polymerase continues to induce in vitro transcription to produce copies of RNA fragments that are subsequently recycled as RNA templates for the synthesis of promoter-bearing dsDNA. However, the formation of promoter-bearing dsDNA has a possibility of failure if the initial low target RNA template copy number in clinical samples is further removed due to ready degradation by the action of host nucleases and/or metal ions (metal-ion-based RNA cleavage). Noticeably, the half-life of exogenous viral RNA in host saliva is approximately 30 s^27^. During this failure event, enzymes and primers are consumed in vain; thus, it takes time for trace amounts of template RNA to enter the amplification cycle due to such failure. To circumvent this issue, we proposed to power the RNA cycle by entering the DNA cycle using RPA, a DNA-specific amplification method that can amplify a target DNA sequence at a constant temperature similar to that used in the RNA-specific amplification method using a recombinase enzyme, a single-stranded DNA-binding protein (SSB), and a strand-displacing polymerase. As of now, we have not come across any study that has reported the co-assisted amplification of RNA and DNA cycles using an isothermal amplification method. In this study, we aimed to advance the bioengineering of our earlier studies on RNA-specific amplification^11,12^ and RPA^8,9,21^ and then integrate the essentials of both types of isothermal amplification methods into a simple format of ‘sample-in and answer-out’ with a primary focus on the detection of low copy numbers of viral RNA directly from COVID-19 saliva samples without the need for any laboratory handling or sample preprocessing. In this regard, we report the development of a completely homogeneous, isothermal, highly sensitive, and ultrarapid method for detecting virus RNA target sequences for the on-site (low resource settings) molecular diagnosis of COVID-19 and other infectious diseases. We named this method the RICCA (RNA Isothermal Co-assisted and Coupled Amplification) reaction. The current manual prototype concurrently measures samples within 15–30 min with a sensitivity as low as a few copies per microliter of living bacterial cells (*E. coli*) and heat-inactivated viruses (SARS-CoV-2 and HCoV-229E).

## Results

### RICCA concept

**Figure 1:**
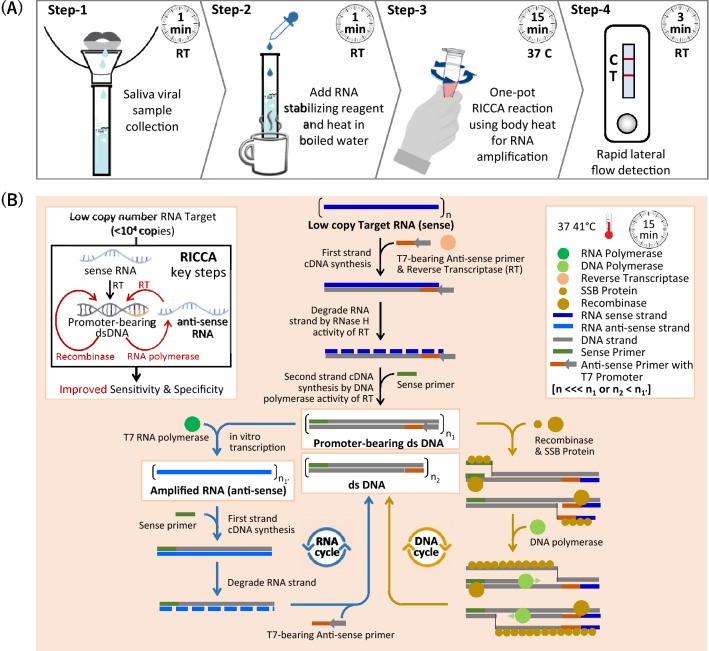
RICCA (RNA Isothermal Co-Assisted Coupled Amplification) test concept and mechanism. (**A**) A working flowchart for the rapid, reliable and field-deployable molecular testing of RNA virus with a sample-to-answer period of less than 20 min using a lateral flow device-powered RICCA test. The four simple operation steps are as follows: a 1-min protocol for easy and painless saliva sampling is used for the collection of biological virus samples (step-1) and for the release of viral RNA (step-2). Next, the lysed solution is directly used for one-pot coupled isothermal RNA and DNA amplification reactions at a temperature near 37 °C for 15–30 min (step-3). Qualitative results are visualized by the naked eye using a DNA lateral flow immunochromatographic assay in 3 min (step 4). (**B**) Schematics of the mechanism and the key steps involved in the RICCA assay to overcome the inevitable challenging features of conventional isothermal amplification methods.

A workflow of the RICCA ‘sample-in and result-out’ approach using a lateral flow (LF) device is shown in Fig. 1A. First, a saliva sample is acquired from the subject using a saliva sample collection tube (e.g., SalivaBio Oral Swab, Salimetrics, LLC.). Second, the direct lysis of virus cells is performed by heating the saliva sample with RNA stabilizing reagent (protease) and/or mixing the saliva sample with detergent (e.g., 1% NP40 or 0.01% Triton X-100) containing buffer (phosphate-buffered saline, pH 7.4). Third, the lysed sample and the RICCA reagents were mixed to initiate a one-pot isothermal nucleic acid amplification reaction near 37 °C using body heat or portable heat block. Finally, the results are visualized by the naked eye using a hand-held DNA LF device. As shown in Fig. 1B, a key concept is the dual-function application of RNA and DNA cycles in the RICCA reaction, including a modified and advanced version of our previously developed RNA-specific amplification method^11,12^, in which the formation of promoter-bearing dsDNA is essential for the template RNA to enter the RNA-specific amplification cycle. This step, however, is the rate-limiting step if the copy number of the target template RNA in the reaction is very low. Furthermore, the possibility of failure in the formation of promoter-bearing dsDNA becomes severe due to the ready degradation of template RNA by the presence of exogenous nucleases in clinical samples such as saliva, which limits the RNA template that can enter the amplification cycle. Therefore, an alternative route for the formation of promoter-bearing dsDNA is essential and is provided in RICCA by the inclusion of RPA, which provides a bypass route and assists RNA amplification, similar to RPA by supplying T7 promoter-bearing dsDNA. Therefore, in RICCA, coupled RNA-specific and DNA-specific amplification reactions co-assist each other by providing a favorable environment (i.e., specific template) to each other and synergistically improve the efficiency of both amplifications, i.e., RNA-specific amplification provides a DNA template to RPA, and RPA provides sufficient template for RNA-specific amplification. In the RICCA protocol, the clinical sample of RNA viruses is added directly into a reaction tube containing all necessary components for the isothermal amplification of RNA-specific amplification and DNA-specific amplification.

### Demonstration of the RICCA concept for one-pot ultrasensitive RNA detection directly from cells

**Figure 2:**
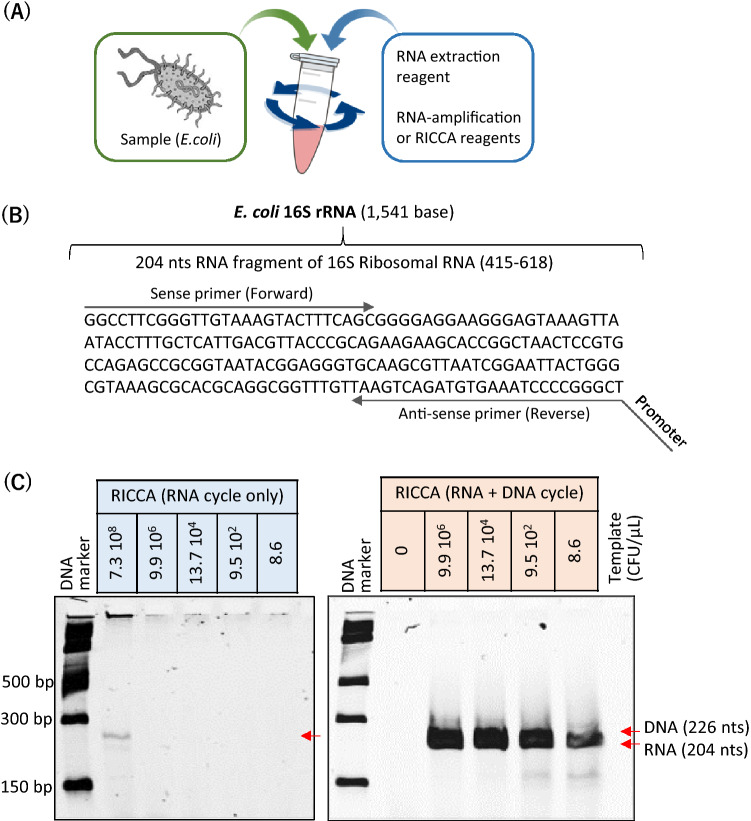
Demonstration of one-pot RICCA for field application. (**A**) Schematic view of the one-pot reaction protocol for RNA extraction to RNA/DNA amplification. (**B**) Target sequence of the *E. coli* genome for amplification. The sequence to which primers bind is indicated by arrows. (**C**) Electrophoretic analysis for comparatively evaluating the detection limits of RNA-specific amplification without (left) and with (right) DNA-specific amplification. *E. coli* cells at levels ranging from 10^9^ to 10^1^ were mixed with saliva (5% v/v) and used directly as the template for RNA extraction followed by RNA or DNA amplification. The desired amplified product (204-nt RNA or 226-bp DNA) is indicated by red arrows.

First, we demonstrated the field-deployable application of a one-pot RICCA reaction for ‘sample-in and answer-out’ testing. For this purpose, an initial assessment of the RICCA platform was performed by detecting RNA directly from nonclinical bacterial cells (i.e., a nonpathogenic strain of living *E. coli*) in the presence of human saliva (Fig. 2A). We designed sequence-specific primers to target a 204-base (or “nt”) RNA fragment of the 16S rRNA gene from the *E. coli* bacterial genome. Figure 2B shows the nucleotide sequence of this fragment. To amplify the antisense RNA, the T7 promoter sequence needed for RNA-specific amplification was added to the reverse primer. We then performed RNA-specific amplification using human saliva spiked with *E. coli* cells as templates in the range between 7.3 × 10^8^ and 8.6 CFU per μL. As shown in Fig. 2C, RICCA with RNA cycle (i.e., RNA-specific amplification only) produced an amplicon of 204 bases only in the reaction with the highest template concentration (7.3 × 10^8^ CFU/μL). However, RICCA with an RNA plus DNA cycle produced amplicons of both RNA and DNA in all reactions, including the lowest template concentration (8.6 CFU/μL). Considering the presence of approximately 20,000 ribosomes in a single cell of *E. coli*, the detection limit was estimated to be less than 10^5^ copies of template RNA in the presence of saliva. This one-pot reaction confirmed the utility of RICCA for field application with ultrahigh sensitivity and within 10 min of the start of the reaction. Importantly, no false-positive (nonspecific) signal was obtained with the RICCA reaction with the negative control sample (no template).

### RNA-specific amplification assay (RICCA with RNA cycle only) for SARS-CoV-2

**Figure 3:**
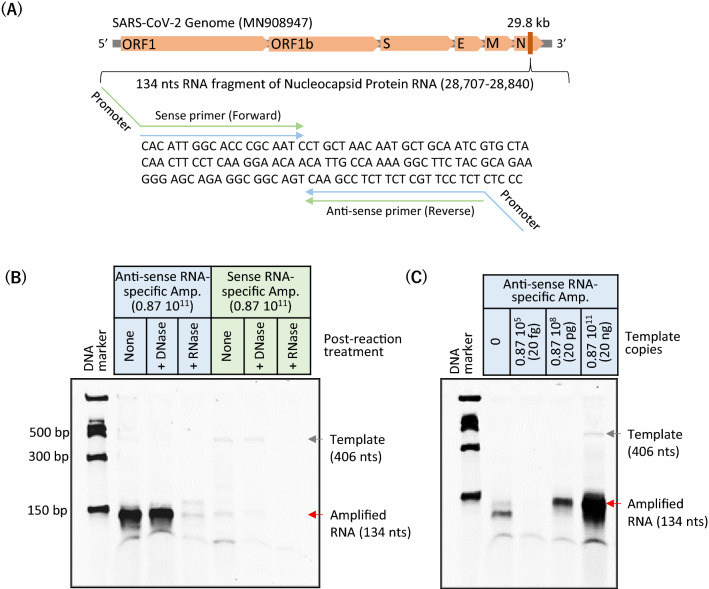
Evaluation of the specificity of RNA-specific amplification of sense or antisense strands of SARS-CoV-2 RNA. (**A**) Structure of the SARS-CoV-2 genome and a target sequence for amplification. The sequence to which primers bind is indicated by arrows. (**B**) Electrophoretic analysis of RNA-specific amplification of antisense or sense strands in 10 min. The desired amplified product (134 nt RNA) is shown by the red arrow and clearly observed with the amplification of the antisense strand of RNA. RNA-specific amplicons were confirmed by postreaction treatment with RNase-free DNase or RNase (RNase ONE and RNase H). (**C**) Electrophoretic analysis for evaluating the detection limit of RNA-specific amplification using SARS-CoV-2 RNA template at a level ranging from 10^11^ to 10^5^ and a zero copy number with a reaction incubation time of 10 min.

To investigate the application of RICCA for the detection of COVID-19, we started by exploring the effect on the efficiency of RNA-specific amplification for amplifying sense or antisense RNA from sense RNA as a template. For this purpose, we compared the sensitivity and specificity of RNA-specific amplification for amplifying the sense or antisense RNA of SARS-CoV-2. We designed sequence-specific primers to target a 134-bp fragment of the nucleocapsid phosphoprotein gene from the SARS-CoV-2 viral genome. As shown in Fig. 3A, the nucleotide sequence of this fragment. Standard RNA (406 bases) was prepared by in vitro transcription using cloned DNA. For the amplification of antisense or sense RNA, the T7 promoter sequence needed for RNA-specific amplification was added to the reverse or forward primer, respectively. We then performed RNA-specific amplification using 20 ng (0.87 × 10^11^ copies) of SARS-CoV-2 RNA. As shown in Fig. 3B, a clear amplicon of 134 bases was observed in the amplification of antisense RNA. The RNA-specific amplicon was further confirmed by postreaction treatment of the amplified product with DNase or RNase. However, this was not the case with the amplification of sense RNA, and a very faint band was observed with the gel electrophoretic analysis. A possible explanation of this failure is demonstrated in SI Appendix, Fig. S1. Based on these results, we chose the primer set to amplify antisense RNA for the RICCA reaction. Noticeably, the appearance of a band of template RNA (406 base) was visible with sense RNA amplification, which indicated the inefficiency of the template for full utilization in the reaction. We then examined the limit of detection (LOD) of RNA-specific amplification of antisense RNA using serial dilutions of 20 ng (0.87 × 10^11^ copies) to 20 fg (0.87 × 10^5^) of SARS-CoV-2 RNA standard template spiked into a 15-μL reaction. As shown in Fig. 3C, RNA-specific amplification produced visible amplicons of 134 bases at decreasing intensities with decreasing template copies. This result demonstrated the lowest LOD of 20 pg (0.87 × 10^8^) within 10 min of the start of the amplification reaction.

### Optimization and sensitivity evaluation of the RNA cycle in RICCA for SARS-CoV-2

**Figure 4:**
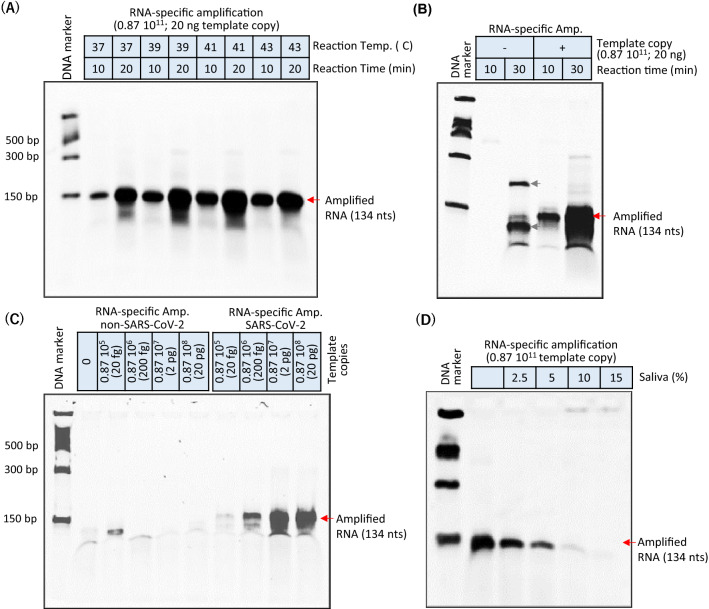
Optimization of antisense strand RNA amplification. (**A**) The effect of temperature in the range of 37–43 °C on the reaction efficiency of RNA amplification was checked at 10 and 20 min. (**B**) The specificity of RNA amplification was evaluated with high (10^11^) or no template copy numbers and shorter (10 min) or longer (30 min) reaction times. (**C**) Evaluation of the selectivity of RNA amplification for the detection of the targeted template (SARS-CoV-2) over a longer template (non-SARS-CoV-2) at a copy number range of 10^5^–10^8^ with a 10-min reaction incubation time. (**D**) The inhibition effect of saliva on the reaction efficiency of RNA amplification was tested in the range of 2.5 to 15% saliva in the reaction. Red and gray arrows indicate true-positive (134 nt) and false-positive amplified products, respectively.

To improve the efficiency of the antisense RNA-specific amplification reaction, we optimized (i) the temperature, (ii) the reaction time, (iii) the selectivity for the targeted template, and (iv) the inhibitory effect of saliva on the reaction efficiency. To test the effect of temperature, we performed RNA-specific amplification at 37 °C, 39 °C, 41 °C and 43 °C for 10 and 20 min each. As shown in Fig. 4A, amplification occurred at all temperatures, and 41 °C appeared to be optimal for the specific target of the SARS-CoV-2 genome. Furthermore, increasing the time from 10 to 20 min increased the yield of amplicons many-fold. Therefore, we further examined the effect of longer reaction times on higher yields. As shown in Fig. 4B, a higher increase in the yield of the specific amplicon was consistently observed after 30 min of reaction. However, nonspecific amplicons of higher and lower molecular weights were observed only with the 30-min reaction if the reaction did not include template. This result is consistent with the fact that nontarget-triggered amplification due to a lower reaction temperature in isothermal amplification is inevitable and magnified with increases in the reaction time. To further investigate this issue, we examined the efficiency of RNA-specific amplification to amplify extremely diluted template copies ranging from 200 fg (0.87 × 10^6^ copies per reaction) to 200 ag (870 copies per reaction or 29 copies/μL) versus longer reaction time. Our results suggested that the yield of a specific amplicon with a lower copy number of template increased with increases in the reaction time but also resulted in nonspecific amplicons; thus, a longer incubation time for the RNA-specific amplification reaction should be avoided, particularly if the sample has a low viral load. Additionally, we noticed that the success of reaction using lower copy number of template (29 copies/μL) was highly depending on the freshly prepared synthetic RNA template and contamination-free experiments since the lower number of smaller size of RNA template can be readily degraded and removed from the system. Subsequently, to examine the primer selectivity for the targeted template of SARS-CoV-2 RNA in RNA-specific amplification, we performed the reaction with a template from a different source (non-SARS-CoV-2). As shown in Fig. 4C, no specific amplicons were observed when the SARS-CoV-2 primers were used to amplify non-SARS-CoV-2 template copies in the range between 20 pg and 20 fg. In contrast and as expected, the analysis of the yield of specific amplicons increased with increases in the template copies of SARS-CoV-2 RNA revealed that the signals of the desired amplicon (134 nt) could be clearly observed using as little as 20 fg (1740 copies/μL) within 10 min of the start of the reaction.

Then, given the bottleneck issues in the handling of nasopharyngeal swabs and with the current growing interest in testing COVID-19 using saliva, we examined whether the presence of digestive enzymes such as RNases in human saliva can readily degrade intact SARS-CoV-2 RNA. Therefore, to adapt RNA-specific amplification to direct saliva samples, we tested the efficiency of RNA-specific amplification in the presence of human saliva. As shown in Fig. 4D, the results indicated that the presence of human saliva in the reaction exerts pronounced inhibitory effects on the RNA amplification process. The inclusion of more than 10% v/v saliva to amplify 20 ng of template RNA induced almost 100% inhibition of the reaction, and minimal inhibition (below 66%) was observed at concentrations below 5% saliva in the RNA-specific amplification reaction. To further improve the sensitivity by utilizing higher concentrations of saliva, we followed the Saliva-Direct approach^28^, which was recently reported to bypass the need for RNA extraction (isolation and purification). Interestingly, we were able to perform successful RNA-specific amplification reactions with the inclusion of a maximum of 10% human saliva using our modified protocol.

### Evaluation of the demonstration of the RICCA concept for SARS-CoV-2

**Figure 5:**
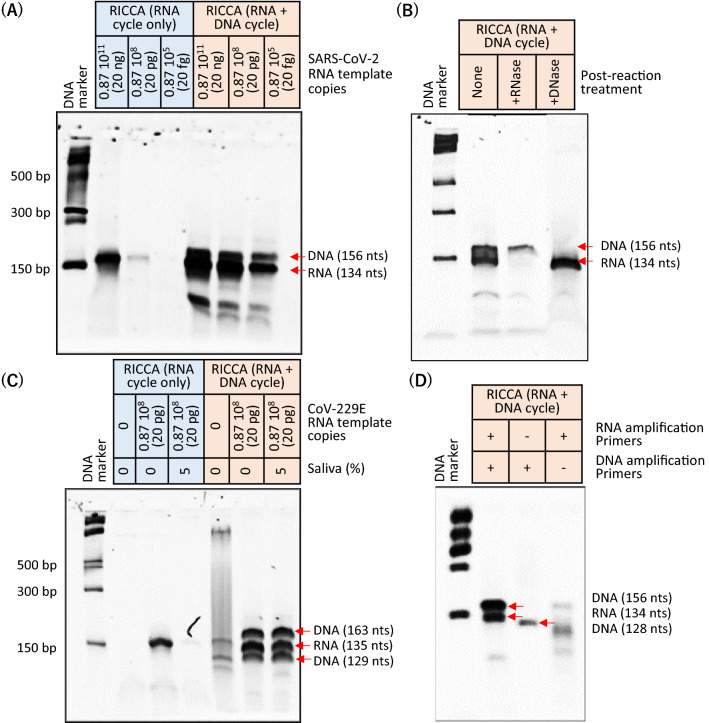
Proof-of-concept demonstration of RICCA. (**A**) Electrophoretic analysis to comparatively evaluate the detection limit of RNA-specific amplification only versus RNA-specific amplification with DNA-specific amplification to amplify low template copy numbers ranging from 20 ng to 20 ag. The desired amplified products (134-nt RNA and 156-bp DNA) are shown by red arrows in the gel electrophoretic analysis. (**B**) RNA- or DNA-specific amplicons were confirmed by treatment with DNase or RNase, respectively. (**C**) Electrophoretic analysis to compare the detection limit of RNA-specific amplification only versus RNA-specific amplification with DNA-specific amplification in the absence or presence of saliva using a freshly synthesized template for CoV-229E RNA. The desired amplified product (135-nt RNA or 163-bp DNA) is indicated by red arrows. (**D**) Electrophoretic analysis to evaluate the co-assisted efficiency of RNA- and/or DNA-specific amplification during the RICCA reaction in the absence and/or presence of primers for RNA and/or DNA-specific amplifications.

To overcome the challenge of low sensitivity in RNA-specific amplification if the template copy number is excessively low (below picograms), we performed a comparative analysis to demonstrate the advantage of the DNA cycle in the RICCA reaction. We sequentially performed reactions by excluding and including a DNA-specific amplification step in the RICCA assay to amplify low template copy numbers ranging from 20 ng (0.87 × 10^11^) to 20 fg (0.87 × 10^5^). A clear difference between RICCA with RNA cycle only and RICCA with RNA plus DNA cycle could be observed by electrophoretic analysis (Fig. 5A). The desired amplicon (134 base) in the electrophoretic analysis was observed using RICCA with an RNA cycle only with high template copies (20 ng); however, for all cases, RICCA with an RNA plus DNA cycle could produce visible amplicons (134-base RNA amplicon and 156-base DNA amplicon) at intensities that decreased with decreases in the template copies. This result demonstrated the lowest LOD for RICCA, and 20 fg (87,000) was amplified within 10 min of the 50 μL-scale reaction start, which corresponds to 1740 template copies per microliter. The efficiency of RICCA for equally producing both DNA-specific and RNA-specific amplicons was further confirmed by postreaction treatment of the amplified product with DNase or RNase, respectively (Fig. 5B). For further evaluation, the amplicon from the RICCA reaction was examined by DNA sequencing and confirmed (S2 Appendix, Fig. S2).

Next, to check the efficiency of RICCA in the presence of saliva, we performed a comparative LOD evaluation for RICCA with RNA cycle only and RICCA with RNA plus DNA cycle in the absence or presence of saliva. For this purpose and to ensure the absence of traces of carryover template contamination, we freshly synthesized a new RNA template for another species of human coronavirus, i.e., CoV-229E, which is one of the viruses responsible for the common cold. As shown in Fig. 5C, electrophoretic analysis confirmed that RNA-specific amplification was not successful only in the case of reaction with saliva; however, including DNA-specific amplification with RNA-specific amplification in RICCA could produce the desired amplified products (135-base RNA and 129-base, 163-base DNA) with no difference between the presence and absence of saliva. This finding surely demonstrates that a co-assisted and coupled approach involving RNA and DNA cycles in RICCA can amplify lower copies of RNA template even in the inhibitory presence of oral saliva at levels as high as 10% v/v in reaction. To further demonstrate and evaluate the co-assisting nature of RNA-specific and DNA-specific amplification, RICCA was performed in the presence and/or absence of both primer sets for both RNA-specific and DNA-specific amplification reactions. As shown in Fig. 5D, RICCA was not successful in the absence of any of the individual primer sets, which further confirms that promoter-bearing second-strand DNA is essential to seed the RICCA reaction. In addition to sensitivity, the specificity of the RICCA test is also an important indicator because the proportion of coinfections is highly expected in COVID-19. Therefore, to examine and ensure the absence of cross-reactivity, primer sets for SARS-CoV-2 and CoV-229E were used for the RNA-specific amplification of CoV-229E and SARS-CoV-2, respectively. The results confirmed the absence of cross-reactivity between SARS-CoV-2 and CoV-229E and indicated the robustness of the RICCA assay, as demonstrated by the very high specificity of the designed primers and template listed in Table 1 (S3 Appendix, Fig. S3).

**Table 1:** Primers and probes used in this study.

	Name	Base position^a^	Sequences (5′ to 3′)
SARS-CoV-2 RNA	Anti-sense strand RNA-specific amplification-134 nts fragment (28,707–28,840)		
	N_Sarbeco_F−	28,706–28,724	CACATTGGCA CCCGCAATC
	T7-N_Sarbeco_R−	28,814–28,833	
	Sense strand RNA-specific amplification-134 nts fragment (28,707–28,840)		
	T7-N_Sarbeco_F+	28,706–28,724	*AATTCTAATACGACTCACTATAGGGAGA*CACATTGGCACCCGCAATC
	N_Sarbeco_R+	28,814–28,833	GAGGAACGAGAAGAGGCTTG
	RICCA reaction-134 nts fragment (28,707–28,840)		
	DIG-N_Sarbeco_F−	28,706–28,724	[Digoxigenin]CACATTGGCACCCGCAATC
	BIO-N_Sarbeco_R−	28,814–28,833	[Biotin]GAGGAACGAGAAGAGGCTTG
	Anti-sense strand RNA-specific amplification- 164 nts fragment (28,195–28,358)		
	2019-nCoV_N1-CDC_F	28,195–28,214	GTTGTTCGTTCTATGAAGAC
	T7-2019-nCoV_N1-CDC_R	28,335–28,358	*AATTOAATACGACTCACTATAGGGAGA*TCTGGTTACTGCCAGTTGARTCTG
	Lateral flow assay probes for 164 nts fragment (28,195–28,358)		
	5DIG-CDC-P1	28,201–28,230	[Digoxigenir]CGTTCTATGAAGACTTTTTAGAGTATCATG
	3-BIOTIN-CDC-P1	28,231–28,260	AAATCTACTTTAGATITTGTTGTGCTTGCA[Biotin]
CoV-229E RNA	Anti-sense strand RNA-specific amplification		
	229E_F	25,151–25,175	TTTTCCGACGTGCTCGAACTTTTTG
	T7-229E_R	25,261–25,285	*AATTCTAATACGACTCACTATAGGGAGA*CGCTCAACAAGGTCACAGTAATGCC
	RICCA reaction		
	DIG-229E_F	25,151–25,175	[Digoxigenin] TTTTCCGACGTGCTCGAACTTTTTG
	BIO-229E_R	25,261–25,285	[Biotin]CGCTCAACAAGGTCACAGTAATGCC
*E. coli* RNA	RNA-specific amplification		
	ECA619_F	0618–0600	AG CCCGG G GATTTCACATC
	T7-ECA415_R	0415–0435	*AATTCTRATACGACTCACTATAGGGAGA*GGCCTTCGGGTTGTAAAGTAC
	RICCA reaction		
	ECA415_F	0415–0442	[Digoxigenin]GGCCTTCGGGTTGTAAAGTACTTTCAGC
	ECA619_R	0618–0593	[Biotin]AGCCCGGGGATTTCACATCTGACTTA

The italicized sequence is the T7 polymerase-binding sequence.

^a^The base position corresponds to that described in the sequence deposited in GenBank.

### Development of a direct saliva-to-RICCA-to-LF assay for lab-free testing of SARS-CoV-2 virus

**Figure 6:**
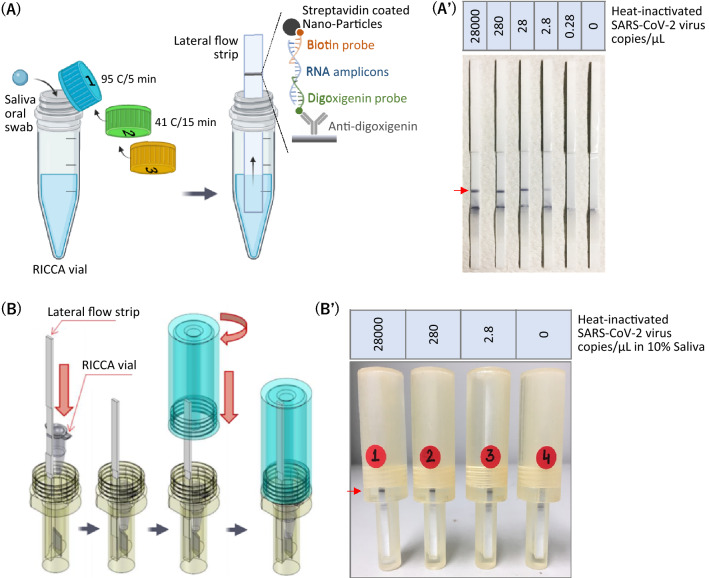
Direct saliva-to-RICCA-to-LF assay for the lab-free testing of SARS-CoV-2 virus. Schematic view of the assay (**A**) and the closed system (**B**). Detection of the heat-inactivated SARS-CoV-2 virus in the absence (**A’**) and presence (**B’**) of 10% human saliva using a lateral flow assay. The true amplified products are indicated by red arrows.

To demonstrate the RICCA approach for lab-free diagnosis, we followed the saliva-direct approach^28^, which was recently reported to bypass the need for an RNA extraction (isolation and purification) step, and a commercial lateral flow (LF) assay that allows rapid and accurate read-out by the naked eye. For the saliva-to-RICCA reaction, we designed a one-pot assay using sequential combinations of multiple components in a one-pot reaction approach. As shown in Fig. 6A, we used a 0.5-mL screw-cap Eppendorf tube with a total of three screw caps consisting of the necessary amounts of RNA stabilizing reagent, RNA amplification reagent and RNA detection reagent to run a single test reaction. For long-term storage, co-dried reagents can be stored in the cap using a glass fiber matrix^29^. For saliva collection, a simple method with a SalivaBio Oral Swab (SOS, Salimetrics LLC, USA) was used^30^. Ten percent human saliva spiked with heat-inactivated SARS-CoV-2 virus was first mixed with RNA stabilizing reagent (cap #1) and heated for 2–5 min at 80–95 °C. Heating at 95 °C demonstrated the remarkable robustness of the procedure. The saliva samples were then mixed thoroughly with RNA amplification reagent (cap #2) by sequentially replacing the caps followed by incubating for 15–30 min at 41 °C. Finally, cap #3 was used to introduce the RNA detection reagent into the reaction mixture. For the LF assay, we designed a pair of digoxigenin-labeled DNA probes and a biotin-labeled DNA probe to enable hybridization with RNA amplicons at the 5′-terminal end and 3′-terminal end, respectively. For high sensitivity, we opted to use an LF assay with a streptavidin patterned line and gold nanoparticle-labeled anti-digoxigenin antibodies for visualization. Subsequently, to demonstrate the RICCA approach for contamination-free (cross-contamination between specimens or synthetically derived PCR amplicon contamination) diagnosis^31^, we aimed to newly design the so-called closed system for the saliva-to-RICCA-to-LF assay, which can avoid aerosol contamination during liquid handling in the LF assay. As shown in Fig. 6B, the RICCA vial and LF strip are placed inside the LF device, and the device is closed by tightening the upper lid. As a result, the bottom side of the RICCA vial approaches an inbuilt blade at the bottom of the LF device and cracks, which allows the RICCA reaction mixture to come out and move up on the LF strip by capillary action. As a result, a positive test reaction showed the appearance of a band (test line) within 3 min for. As shown in Fig. 6A’, RNA amplicons yielded a clear band in the RICCA-to-LF assay with ultrahigh sensitivity, and a concentration as low as 2.8 copies/μL of SARS-CoV-2 virus could be detected. We then demonstrated the application of a closed system using an LF device and tested the sensitivity in the presence of 10% human saliva (Fig. 6B’). Interestingly, we found that the result from the direct saliva-to-RICCA-to-LF assay is fortunate and important and exhibits high sensitivity with levels as low as 2.8 copies/μL of SARS-CoV-2 virus (true-positive control reaction) and high specificity (true-negative control reaction). We further confirmed the results by analyzing the RNA amplicons with a size of 164 nt by gel electrophoresis (S4 Appendix, Fig. S4). These results demonstrate the remarkable simplicity and robustness of the RICCA assay for the lab-free and rapid testing of COVID-19.

## Discussion

We introduced and demonstrated a robust isothermal amplification assay, termed the RICCA, that enables a simple one-pot ‘sample-in and answer-out’ format for the ultrarapid detection of low copy numbers of RNA virus directly from saliva, with a total assay time within 30 min and with a sensitivity as low as few copies of SARS-CoV-2 virus per microliter. The RICCA assay requires only a heat block and a tube prefilled with a mixture containing the necessary reagents (substrate, primer, and enzyme) to simultaneously perform RNA-specific and DNA-specific amplifications. We estimated that the material cost for in-house reagents for RICCA is very affordable (RNA-specific amplification reagents cost USD 2.6 per reaction, and DNA-specific amplification reagents cost USD 1.3 per reaction); thus, RICCA has the potential to decentralize COVID-19 molecular diagnosis to realize a ‘lab-free, lab-quality’ mega-testing platform. In order to evaluate the rapidity of RICCA, a real-time analysis was used to assess the performance of real-time RICCA in a reference with conventional real-time RT-PCR approach (S5 Appendix, Fig. S5). A cycle threshold (Ct) value for RICCA was significantly faster than Ct value for RT-PCR. These results indicated that the RICCA reaction speed was excellent for rapid diagnosis. In conclusion, we have devised a novel approach RICCA using an improved isothermal RNA amplification method. In order to show the potential of RICCA to contribute to COVID-19 diagnosis, early phase clinical trials have already been performed at hospitals in India and Japan and trials using a large number of clinical samples are now in the planning. Following this, we hypothesized that a robotic and mobile platform equipped with an automatic dispenser unit (a desk-top prototype is under development) for RICCA could be applied to taking COVID-19 diagnostics directly to consumers by mitigating the logistical burden of sample transport and could provide large-scale population testing in remote or resource-limited settings. More importantly, this platform could facilitate the expansion of COVID-19 testing to other infectious viral or bacterial strains with only an additional step of primer design.

## Methods

### Preparation of standard SARS-CoV-2 or CoV-229E RNA templates

The sequence of the SARS-CoV-2N gene was identified from GenBank (accession number MN908947), and the 134-nt single-stranded DNA fragment of the nucleocapsid protein gene, corresponding to the RNA sequence at position 28,707–28,840, was synthesized in vitro. A T7 RNA polymerase promoter sequence was added to the 5’ terminal end, and the double-stranded DNA construct was synthesized by overlap extension PCR using DNA oligomers (Table 1) purchased from a commercial supplier (Eurofins Genomics, Tokyo, Japan). The RNA transcript was synthesized using a cell-free transcription kit (RiboMAX, Promega, Madison, WI, USA) and purified (RNeasy, Qiagen, Tokyo, Japan). The concentration of purified RNA was determined spectrophotometrically (Nanodrop ND-2000, Thermo Fisher Scientific, Waltham, MA), and standard templates ranging from 10^9^ to 1 RNA copies/μL were serially diluted and freshly prepared in Tris–EDTA (TE) buffer.

### Preparation of standard *Escherichia coli* rRNA template

The sequence of *Escherichia coli* (*E. coli*) 16S rRNA was identified from GenBank (accession number J01859.1), and the 204-nt single stranded DNA fragment of 16S rRNA, corresponding to the RNA sequence at position 415–618, was synthesized in vitro. The test strain (NBRC 3301) of nonpathogenic *E. coli* was obtained commercially (NITE Biological Resource Center, Tokyo, Japan) and grown in L broth at 37 °C for 24 h. The cell counts (CFUs per mL) in serially diluted bacterial culture were determined using a JuLI-smart fluorescent cell analyzer and conventional hemocytometer. For isothermal amplification reactions, cells were harvested from 1 mL of culture by 5 min of centrifugation at 15,000×*g*, and RNA extraction was performed by disrupting the bacterial cells first by sonication and then by treatment with NP40 reagent (Surfact-Amps NP40, Thermo Fisher Scientific). The solution was then centrifuged at 15,000×*g* for 5 min to sediment cell debris. The supernatant was used directly as a template for isothermal amplification reactions or was stored at − 20 °C until use.

### RNA-specific amplification assay

The reaction mixture was prepared by mixing 5.0 μL of substrate stock solution [17.6 μL of nuclease-free water (NFW), 5.6 μL of 1 M MgCl_2_, 19.8 μL of 1.9 M Tris HCl (pH 8.6), 0.6 μL of 40 U/μL RNase Inhibitor, 3.3 μL of 100 mM DTT, 41.3 μL of 2.0 mM dNTP (each), 17.1 μL of 58 mM NTP (each) and 4.8 μL of 250 mM inosine triphosphate], 5.0 μL of primer stock solution [32.5 μL of NFW, 21.5 μL of 2 M KCl, 6.6 μL of 50 μM sense forward primer, 6.6 μL of 50 μM promoter-bearing antisense reverse primer and 42.9 μL of dimethylsulfoxide] and 2.5 μL of RNA template in a PCR tube and incubated at 65 °C for 5 min (optional) and 41 °C for 5 min. The reaction was started by adding 2.5 μL of enzyme stock solution [4.0 μL of 10 mg/mL Bovine Serum Albumin, 4.4 μL of 20,000 unit/mL AMV Reverse Transcriptase (Life Sciences Inc, Petersburg, FL), 11.1 μL of 60% sorbitol and 35.5 μL of T7 RNA Polymerase of 50,000 unit/mL (Toyobo)] and continued at 41 °C for 10 to 30 min. The amplified products were analyzed using 1-inch 6% denatured polyacrylamide gel^24^ and SYBR Gold staining.

### RNA-specific amplification assay with DNA-specific amplification

The reaction was performed using a TwistAmp Basic kit (TwistDx, Cambridge, MA, USA). Briefly, the reaction mixture for RNA-specific amplification (5.0 μL of substrate stock solution, 5.0 μL of primer stock solution, and 2.5 μL of standard RNA or sample) was mixed with 5 μL of primer mix (10 μM digoxin-modified sense/forward primer and 10 μM biotin-modified antisense/reverse primer) and 29.5 μL of primer-free rehydration buffer. The resultant 49.5 μL of mixture and 2.5 μL of enzyme stock solution were added to the dried enzyme pellet supplied by the TwistAmp Basic kit. The reaction was initiated by adding 2.5 μL of 280 mM magnesium acetate and immediately placed at 41 °C for 10 min. The amplified products were analyzed using a 1-inch 6% denatured polyacrylamide gel^24^ and SYBR Gold staining.

### Gel electrophoretic analysis

A 1-inch electrophoretic device developed in-house was used for gel-based detection^24^. Ten-well microcassettes with 6% T polyacrylamide gel were prepared using 40 (w/v)%-acrylamide/bis (19:1) (Nacalai Tesque, Kyoto, Japan), 5× TBE, 10% ammonium persulfate (Fujifilm Wako Pure Chemicals) and tetramethylethylenediamine (Fujifilm Wako Pure Chemicals, Osaka, Japan) and used as a 1-inch (2.5 × 2.5 cm) precast polyacrylamide gel to analyze the RNA-specific amplification or RICCA reaction products. Paper pads (1.0 × 2.0 cm) were soaked in 2 mL of 1× TBE buffer and used as a source of running buffer. One microliter of each sample was loaded in the micro gel wells with 0.5 µL of 6× gel loading dye. A 50-bp DNA ladder was also loaded in one well to confirm the size of the obtained DNA fragments and comigrated with the samples at 100 V for 5–7 min. A 300-μL volume of 10× SYBR gold nucleic acid gel stain (Thermo Fisher Scientific) was poured on top of the gel, and the bands were visualized using the blue LED flashlight installed in the palm-sized electrophoretic device. Images were acquired using a standard camera-equipped cellular phone.

### Real-time analysis

The real-time RICCA or PCR assay was performed with a Thermal Cycler Dice Real Time System (Thermo Fisher Scientific). The real-time RICCA reaction was prepared in a 50-μL reaction volume as described in an earlier section with the addition of 800 × SYBR™ Green I dye (Invitrogen). For real-time PCR, the first reverse transcription reaction was performed using MM4 reverse transcriptase^32^. The composition of the 20-μL reaction volume was as follows: 2 μL of 10 nM MM4 enzyme, 1 µL of 1 mM dNTP mixture, 2 µL of 10 × RT buffer, 0.5 µL of RNase inhibitor (20 U), 1 µL of RNA template, and 3.5 µL of nuclease-free water. The reaction program was 42 °C for 30 min followed by incubation at 65 °C for 5 min. Real-time PCR was conducted using the TB Green™ Premix Ex Taq™ II Kit (Tli RnaseH Plus, Takara Bio, Kusatsu, Japan) according to the manufacturer’s instructions. The composition of the 25-μL reaction volume was as follows: 12.5 μL of 2 × SYBR Premix Ex Taq II (Tli RNaseH Plus), 1 µL of forward primer (10 µM), 1 µL of reverse primer (10 µM), 2 µL of template (reverse transcription product), and 8.5 µL of nuclease-free water. The following thermal program was used: reverse transcription at 55 °C for 15 min followed by 90 °C for 30 s and 35 cycles of amplification (30 s at 94 °C, 30 s at 60 °C, and 30 s at 72 °C).

### Lateral flow immunochromatography assay

The lateral flow dipstick (LFD) was provided by Asahi Kasei Pharma, Japan. The amplification products from the RICCA reaction were added to the sample pad of the LFD strip and allowed to migrate by capillary action, and during process, the biotinylated RICCA product hybridized with a digoxin-labeled DNA probe and complexed with a gold-labeled anti-digoxin antibody. This hybridization product was trapped by a biotin ligand and bound to a lateral flow test strip to form an immune complex bound at the test line (T). The experimental procedure and determination method were as follows: 5 μL of RICCA product was added to 95 μL of NFW, the mixture was added to the sample mat on the test paper strip, and the result was read after 5–10 min.

### Direct saliva-to-RICCA-to-LF assay

Heat-inactivated SARS-CoV-2 was obtained from ATCC (ATCC VR-1986HK; American Type Culture Collection, Manassas, VA, USA). The concentration of virus in lot #70037781 was estimated by the preinactivation titer (6.45 × 10^6^ TCID50/mL), and the RNA copy number was determined by ddPCR (4.2 × 10^5^ genome copies/μL). Prior to the RICCA reaction, the stock aliquot vial was centrifuged and serially diluted in phosphate-buffered saline (PBS) with replicates of 42,000, 4,200, 420, 42, and 4.2 copies/μL. Two microliters of diluted heat-inactivated SARS-CoV-2 virus was spiked with and without 3 μL of human saliva, which was freshly collected using a SalivaBio Oral Swab (SOS, Salimetrics LLC, USA). The 5-μL SARS-CoV-2 spiked samples were mixed with 0.5 μL of Proteinase K (Takara) in 0.2-mL nuclease-free and thin-walled microcentrifuge tubes by simple tapping or vortexing followed by heating in a portable heat block (Mini-Block Bath, MD-Mini, AXEL, Japan) at 95 °C for 5 min. To the lid of the tube, 10 μL of substrate stock solution, 10 μL of primer stock solution and 5 μL of enzyme stock solution were added, and the vial was then spun down the vial and incubated at 41 °C for 15–30 min. Of the 30-μL reaction sample, 28 μL was utilized for the lateral flow (LF) assay, and 2 μL of the sample was used for gel electrophoresis analysis. To prevent possible contamination during liquid handling in the LF assay, we introduced a closed system by designing a device to continuously perform the RICCA reaction and LF assay without opening the RICCA tube. To this end, a prototype is fabricated using a 3D printer that can hold RICCA tubes and LF assay strips. In this design, after a tube is inserted and mechanically forced down by closing the device, a crack forms in the bottom of the tube, and the reaction solution flows out and moves up on the LF strip by capillary action. For the LF assay, 30 μL of the RICCA product was mixed with RNA detection reagents (1 μL of each 20 μM LF assay probe (Table 1), 15 μL of 1 M KCl, and 53 μL of nuclease-free water) in a 1.5-mL microcentrifuge tube, and the mixture was then incubated at 60 °C for 2 min. An LF strip was inserted in the tube, and the test line was observed within 3 min.

## Supplementary Information

Supplementary Figures.
