# Supplementary material for: Development of robust isothermal RNA amplification assay for lab-free testing of RNA viruses
Source: Sci Rep. 2021 Aug 6;11:15997. doi: 10.1038/s41598-021-95411-x (PMC8346491; doi:10.1038/s41598-021-95411-x)

**Supplementary Information**

**Development of robust isothermal RNA amplification assay for lab-free testing of RNA viruses**

Radhika Biyani^a,#^, Kirti Sharma^b,#^, Kenji Kojima^c^, Madhu Biyani^b,d^, Vishnu Sharma^d^, Tarun Kumawat^d^, Kevin Maafu Juma^c^, Itaru Yanagihara^e^, Shinsuke Fujiwara^f^, Eiichi Kodama^g^, Yuzuru Takamura^a^, Masahiro Takagi^a^, Kiyoshi Yasukawa^c^, and Manish Biyani^a,b,d,*^

^a^Department of Bioscience and Biotechnology, Japan Advanced Institute of Science and Technology, 1-1 Asahidai, Nomi City, Ishikawa 923-1292, Japan

^b^BioSeeds Corporation, JAIST Venture Business Laboratory, Ishikawa Create Labo, Asahidai 2-13, Nomi City, Ishikawa 923-1211, Japan

^c^Division of Food Science and Biotechnology, Graduate School of Agriculture, Kyoto University, Sakyo-ku, Kyoto 606-8502, Japan

^d^Biyani BioSolutions Pvt. Ltd., Biyani Group of Colleges Venture Business Laboratory, R-4, Sector 3, Vidhydhar Nagar, Jaipur 302039, India

^e^Department of Developmental Medicine, Research Institute, Osaka Women’s and Children’s Hospital, 840 Murodocho, Izumi, Osaka 594-1101, Japan

^f^Department of Bioscience, School of Biological and Environmental Sciences, Kwansei-Gakuin University, 2-1 Gakuen, Sanda, Hyogo 669-1337, Japan

^g^Division of Infectious Diseases, International Research Institute of Disaster Science, Tohoku University, 2-1 Seiryocho Aoba-ku, Sendai, Miyagi 980-8575, Japan

*To whom correspondence may be addressed. E-mail: [biyani@jaist.ac.jp](mailto:biyani@jaist.ac.jp) or [drmanishbiyani@gmail.com](mailto:drmanishbiyani@gmail.com)

^#^These authors contributed equally to this work.

**Supplementary Figure S1:** Schematics of the mechanism and the key steps involved in RNA-specific amplification. The formation of promoter-bearing dsDNA which is essential for the template RNA to enter into the RNA-specific amplification cycle is synthesized inefficiently while targeting the synthesis of sense RNA strand from sense RNA template (shown by red arrows).

**
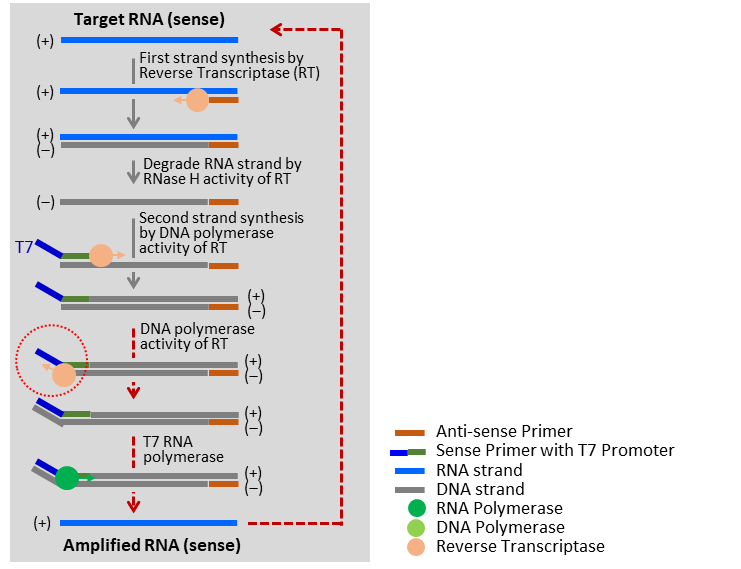
**

**Supplementary Figure S2:** The sequence alignment of the SARS-CoV-2 RICCA amplicon with the actual source of template SARS-CoV-2. The RICCA amplicon was amplified using 20 ag template copy number.

**
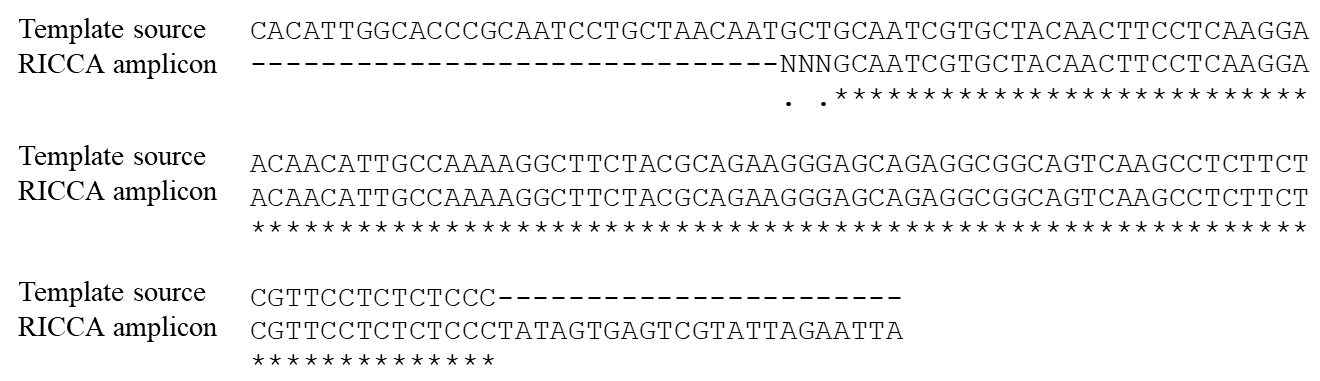
**

**Supplementary Figure S3**: Electrophoretic analysis to check cross-reactivity in RNA-specific amplification using template and primer sets of SARS-CoV-2 and/or CoV-229E. (A) Structure of CoV-229E genome and a target sequence for amplification. The sequence to which primers bind are indicated by arrows. (B) Electrophoretic analysis of RNA-specific amplification of anti-sense amplicon in 10 minutes.

**
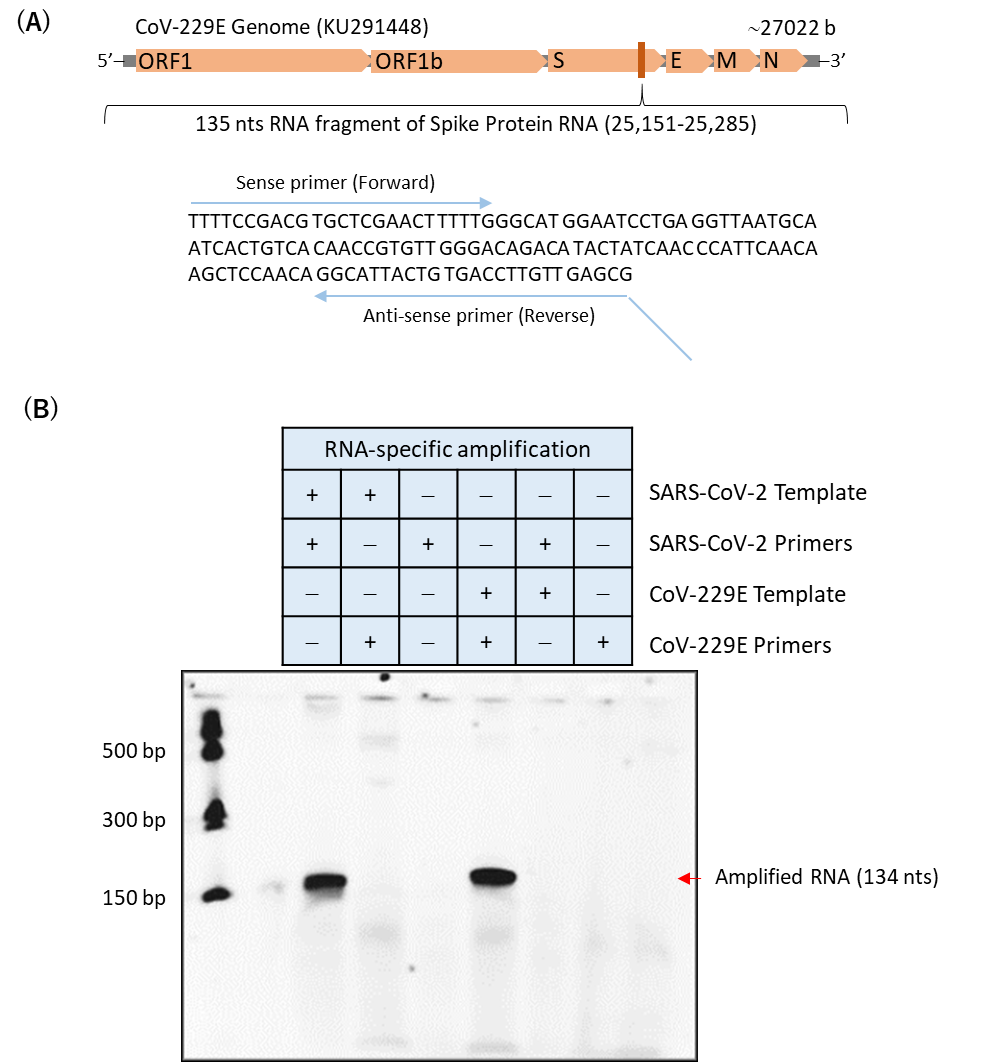
**

**Supplementary Figure S4: Electrophoretic analysis to check sensitivity and specificity of direct saliva-to-RICCA assay.** Electrophoretic analysis to comparatively evaluate the detection limits of RNA-specific amplification of SARS-CoV-2 virus in absence (A) and presence (B) of 10% human saliva in 30 minutes of reaction time. (C) The effect of reaction time on the reaction efficiency of RNA amplification with and without SARS-CoV-2 virus template was checked at 15 and 30 min.

**
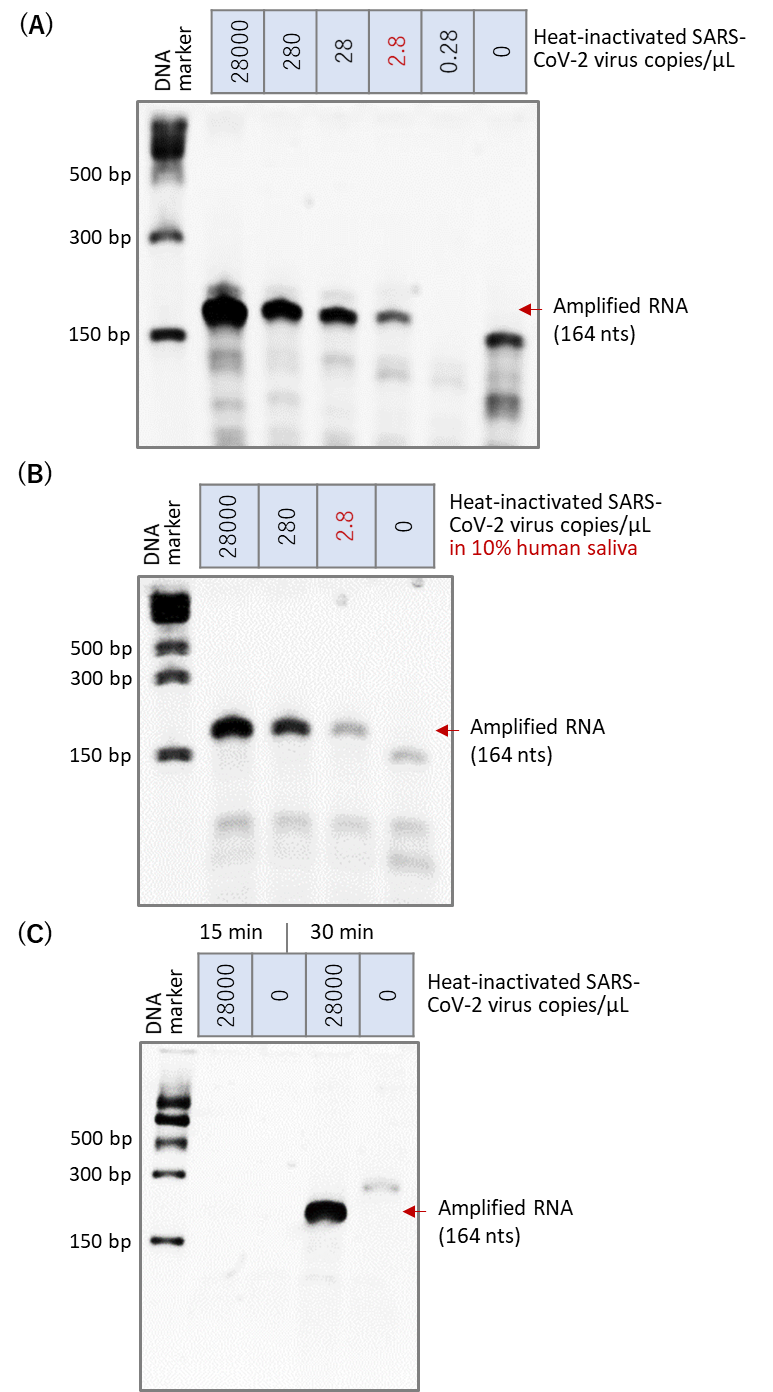
**

**Supplementary Figure S5: Comparative analysis of real-time amplification of the RT-PCR and RICCA reaction.** RT-PCR and RICCA were performed with (20 pg template for CoV-229E RNA) or without template. To determine the reaction efficiency (rapidity), the Ct values are plotted against the reaction time. (A) A Ct value for RICCA was observed at a threshold time near 3 min, which was significantly 7-times faster than RT-PCR (threshold time near 21 min). (B) The end products for all the reactions were also confirmed by gel electrophoretic analysis.


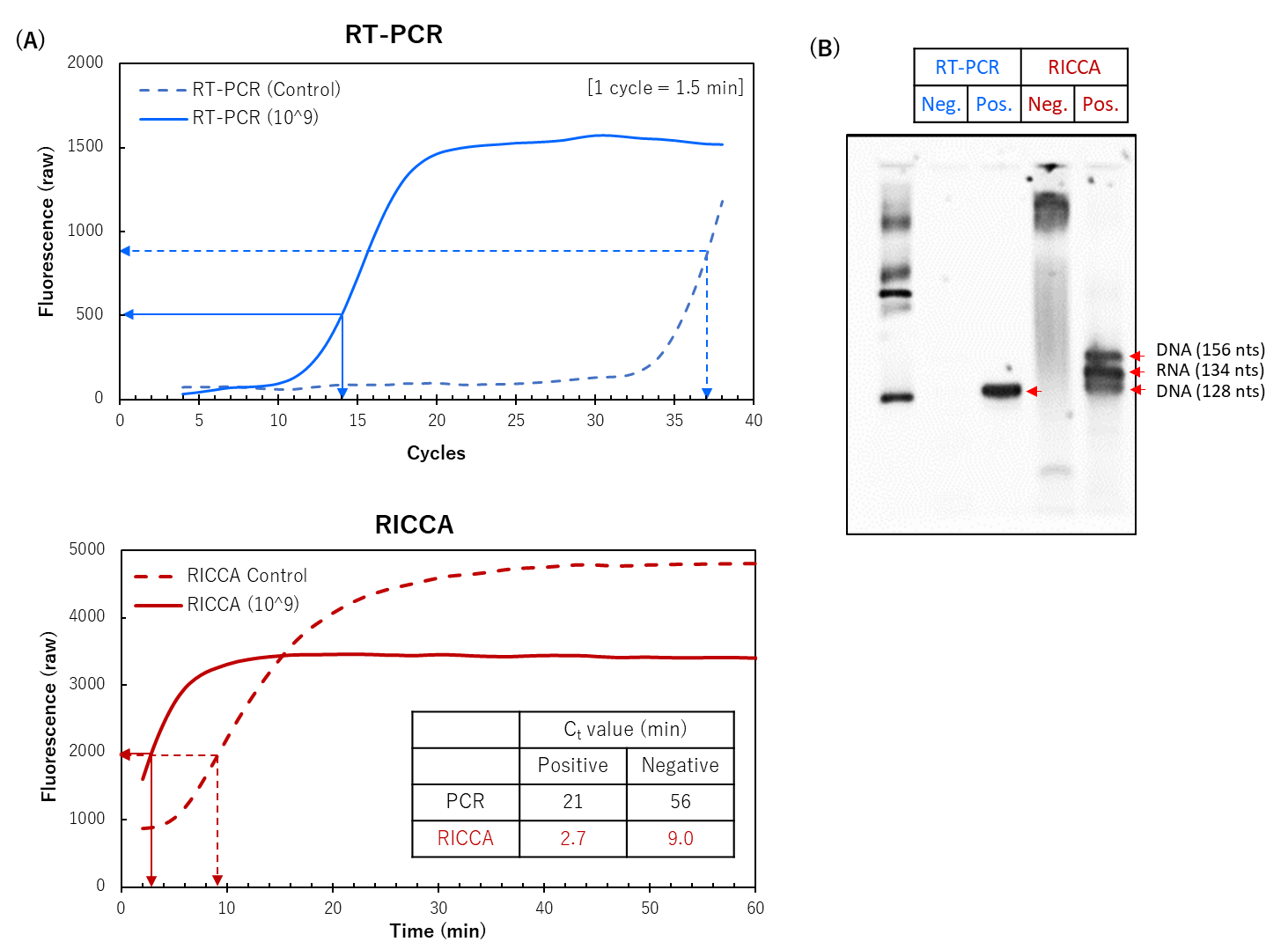

Supplement: Supplementary file 1 — Supplementary Figures. [file 41598_2021_95411_MOESM1_ESM.docx]
